# System-Driven and Oscillator-Dependent Circadian Transcription in Mice with a Conditionally Active Liver Clock 

**DOI:** 10.1371/journal.pbio.0050034

**Published:** 2007-01-30

**Authors:** Benoît Kornmann, Olivier Schaad, Hermann Bujard, Joseph S Takahashi, Ueli Schibler

**Affiliations:** 1 Department of Molecular Biology, University of Geneva, Geneva, Switzerland; 2 Department of Biochemistry, University of Geneva, Geneva, Switzerland; 3 Zentrum für Molekulare Biologie, Universität Heidelberg, Heidelberg, Germany; 4 Howard Hughes Medical Institute, Department of Neurobiology and Physiology, Northwestern University, Evanston, Illinois, United States of America; University of Cambridge, United Kingdom

## Abstract

The mammalian circadian timing system consists of a master pacemaker in neurons of the suprachiasmatic nucleus (SCN) and clocks of a similar molecular makeup in most peripheral body cells. Peripheral oscillators are self-sustained and cell autonomous, but they have to be synchronized by the SCN to ensure phase coherence within the organism. In principle, the rhythmic expression of genes in peripheral organs could thus be driven not only by local oscillators, but also by circadian systemic signals. To discriminate between these mechanisms, we engineered a mouse strain with a conditionally active liver clock, in which REV-ERBα represses the transcription of the essential core clock gene *Bmal1* in a doxycycline-dependent manner. We examined circadian liver gene expression genome-wide in mice in which hepatocyte oscillators were either running or arrested, and found that the rhythmic transcription of most genes depended on functional hepatocyte clocks. However, we discovered 31 genes, including the core clock gene *mPer2,* whose expression oscillated robustly irrespective of whether the liver clock was running or not. By contrast, in liver explants cultured in vitro, circadian cycles of *mPer2::luciferase* bioluminescence could only be observed when hepatocyte oscillators were operational. Hence, the circadian cycles observed in the liver of intact animals without functional hepatocyte oscillators were likely generated by systemic signals. The finding that rhythmic *mPer2* expression can be driven by both systemic cues and local oscillators suggests a plausible mechanism for the phase entrainment of subsidiary clocks in peripheral organs.

## Author Summary

In contrast to previously held belief, molecular circadian oscillators are not restricted to specialized pacemaker tissues, such as the brain's suprachiasmatic nucleus (SCN), but exist in virtually all body cells. Although the circadian clocks operative in peripheral cell types are as robust as those residing in SCN neurons, they quickly become desynchronized in vitro due to variations in period length. Hence, in intact animals, the phase coherence between peripheral oscillators must be established by daily signals generated by the SCN master clock. Although the hierarchy between master and slave oscillators is now well established, the respective roles of these clocks in governing the circadian transcription program in a given organ have never been examined. In principle, the circadian expression of genes in a peripheral tissue could be driven either by cyclic systemic cues, by peripheral oscillators, or by both. In order to discriminate between genes regulated by local oscillators and systemic cues in liver, we generated mice in which hepatocyte clocks can be turned on and off at will. These studies suggest that 90% of the circadian transcription program in the liver is abolished or strongly attenuated when hepatocyte clocks are turned off, indicating that the expression of most circadian liver genes is orchestrated by local cellular clocks. The remaining 10% of cyclically expressed liver genes continue to be transcribed in a robustly circadian fashion in the absence of functional hepatocyte oscillators. These genes, which unexpectedly include the bona fide clock gene *mPer2,* must therefore be regulated by oscillating systemic signals, such as hormones, metabolites, or body temperature. Although temperature rhythms display only modest amplitudes, they appear to play a significant role in the phase entrainment of *mPer2* transcription.

## Abstract 3

Research on mice engineered with an inducible liver clock enabled identification of some genes with expression controlled by the local clock, and other genes (including*mPer2*) that maintained circadian oscillations thanks to cues from the SCN.

## Introduction

In mammals, virtually all body cells possess self-sustained, cell-autonomous circadian clocks [1–3]. The oscillators in peripheral organs are entrained by a master pacemaker residing in the suprachiasmatic nucleus (SCN) of the brain's hypothalamus, which is itself synchronized by daily light–dark cycles [4]. The molecular details of the signaling pathways used by the SCN to phase-entrain peripheral clocks are still obscure; however, daily feeding–fasting cycles, circadian hormones, and body temperature appear to play pivotal roles in this process [5–9]. The accumulation of mPER1 and/or mPER2, two integral clock components, is altered upon the administration of phase-shifting cues. Hence, these proteins are likely to be involved in the synchronization of circadian clocks [10,11].

On the molecular level, mammalian circadian oscillators are thought to rely on two interconnected negative loops of clock gene expression [12,13]. According to this model, the principal feedback loop is driven by the repressors PER1, PER2, CRY1, and CRY2 and the PAS-domain basic helix-loop-helix (PAS-bHLH) transcription factors BMAL1, CLOCK, and probably NPAS2 [14]. The transcription of the repressor-encoding genes is activated by these PAS-bHLH transcription factors until the PER-CRY complexes reach critical concentrations at which they annul the transactivation potential of the PAS-bHLH proteins and thereby inhibit transcription of their own genes. The concentration of PAS-bHLH activators is adjusted by an accessory feedback loop in which the orphan nuclear receptor REV-ERBα (and, probably to a lesser extent, its paralog REV-ERBβ) periodically represses *Bmal1* transcription. The inhibitory activity of REV-ERBα counteracts the transactivation activity of ROR nuclear orphan receptors, which bind to the same RORE elements within the *Bmal1* promoter [15]. The cyclic expression of REV-ERBα is itself governed by the PAS-bHLH activators and CRY-PER repressors of the principal negative feedback loop, thereby interconnecting the *Rev-erbα*-*Bmal1* feedback loop directly to the principal feedback loop [16,17]. Since BMAL1 and CLOCK are metabolically more stable than CRY and PER proteins, their abundance varies only slightly throughout the day [16,18,19].

Post-translational protein modifications are also believed to play important roles in the modulation of PER and CRY activities [18,20,21]. However, to date, *Bmal1* is the only known clock gene whose inactivation immediately leads to arrhythmicity of behavior and to the ablation of *mPer1* and *mPer2* mRNA accumulation cycles in the SCN [22].

Transcriptome profiling studies have uncovered a large number of cyclically expressed genes [23–27]. Although most of these genes appear to be involved in output functions, some may also serve as input regulators participating in the synchronization of local clocks. The oscillating activity of these latter genes would be expected to integrate systemic cues such as circadian hormones, metabolites, or body temperature rhythms, into the clockwork circuitry of peripheral cell types. In the intact organism, the cyclic expression of such genes should not necessarily depend upon functional local clocks.

In this study, we describe our attempt to engineer a mouse strain with conditionally active hepatocyte circadian clocks. We used these mice to classify mRNAs into transcripts whose circadian accumulation in the liver do or do not require local circadian oscillators. The identification of systemically driven and oscillator-driven genes not only provides insight into the structural organization of the mammalian circadian timing system, but should also open new avenues to study the phase entrainment mechanisms of peripheral clocks.

## Results

### The Construction of Mice with a Conditionally Active, Liver-Specific Rev-erbα Transgene

**Figure 1:**
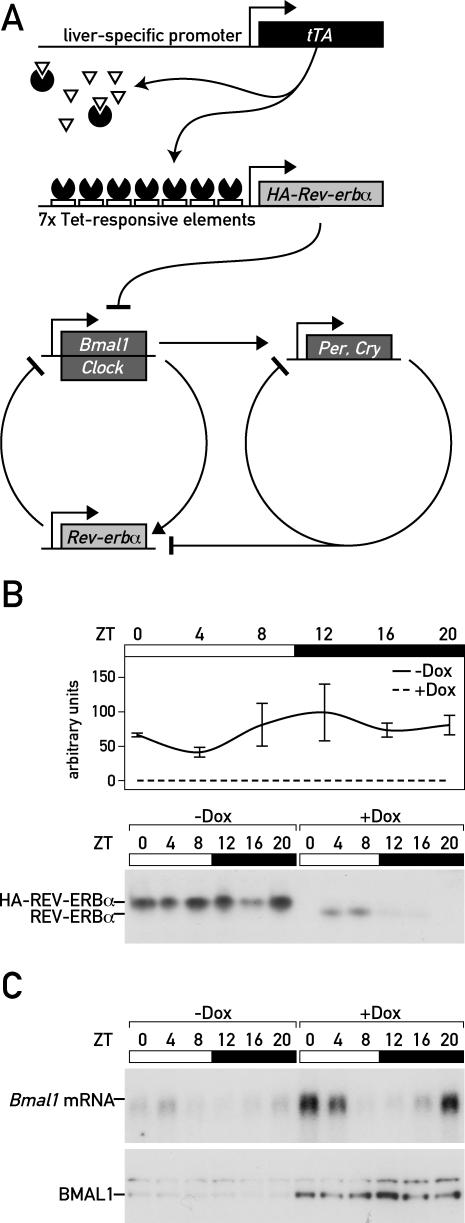
Conditional Repression of *Bmal1* Transcription in Hepatocytes (A) Hepatocyte-specific, Dox-dependent expression of HA-REV-ERBα was achieved by placing a 5′-HA-tagged REV-ERBα cDNA transgene under the control of seven TREs (Tet-responsive elements). In the liver of mice expressing the tetracycline (Tet)-responsive transactivator (tTA) from the hepatocyte-specific *C/ebpβ-LAP* locus control region, *HA-Rev-erbα* transcription is constitutively repressed in the absence of the tetracycline analog Dox (tet-off system). This leads to an attenuation of circadian oscillator function, since *Bmal1* is required for circadian rhythm generation. (B) *LAP-tTA*/*TRE-Rev-erbα* double transgenic mice display high *HA-Rev-erbα* mRNA and protein levels throughout the day in the absence of Dox (−Dox). In the presence 3 g/kg Dox in the food (+Dox), neither *HA-Rev-erbα* mRNA nor protein can be detected. (C) The levels of both *Bmal1* mRNA and BMAL1 protein are dramatically down-regulated in the absence of Dox (compare the lanes on the left to those on the right).

We wished to engineer a mouse strain with conditionally active circadian oscillators specifically in hepatocytes, in order to examine the contribution of local clocks and systemic Zeitgeber cues to rhythmic liver gene expression. As mentioned above, BMAL1 is a constituent of the molecular oscillator whose loss of function immediately results in the abolishment of all manifestations of circadian physiology and gene expression [22]. We thus thought that the conditional expression of *Bmal1* specifically in hepatocytes may provide such a model system. *Bmal1* transcription follows a high-amplitude circadian cycle, owing to the circadian accumulation of REV-ERBα, a nuclear orphan receptor that strongly represses *Bmal1* transcription (Figure 1A) [16]. We thus exploited this regulatory mechanism to produce a mouse strain with a conditionally active *Bmal1* gene in hepatocytes. First, a transgenic mouse strain was established in which transcription of an HA epitope-tagged REV-ERBα version (HA-REV-ERBα) is controlled by tetracycline-responsive elements (TREs). These mice were crossed with *LAP-tTA* mice, which express a tetracycline-dependent transactivator (tTA) specifically in hepatocytes [28]. In *LAP-tTA*/*TRE-Rev-erbα* double transgenic mice, HA-REV-ERBα accumulated to constitutively high levels and suppressed *Bmal1* expression throughout the day in the absence of the tetracycline analog doxycycline (Dox) (Figures 1B, 1C [left panel], and S1). *LAP-tTA*/*TRE-Rev-erbα* mice thus produced HA-REV-Erbα in a liver-specific and tetracycline-dependent fashion. In the presence of Dox, the *TRE-Rev-erbα* transgene remained silent, and circadian oscillator function was not perturbed in liver cells (Figures 1B, 1C [right panel], and S1). As shown in Figure 1B, the regulation of *TRE-Rev-erbα* transgene expression was exquisitely tight, since in Dox-fed mice, neither mRNA nor HA-REV-ERBα protein was detectable by Taqman RT-PCR assays and Western blot experiments, respectively. At least in part, the low levels of *Bmal1* mRNA and protein observed in the liver of untreated animals may have been contributed by endothelial cells, bile duct cells, or Kupffer cells, which did not express the *LAP-tTA* transgene [28]. In rat liver, nonparenchymal cells contribute about 35% of all cells and about 10% of the cellular hepatic volume [29,30], and we thus expect that around 10% of the liver RNA is contributed by cells that do not express the *LAP-tTA* transgene.

We have used mice homozygous for both the *LAP-tTA* and *TRE-Rev-erbα* transgenes in these experiments, in order to maximize transgene expression. Double homozygous mice were born at Mendelian ratios and did not show any phenotype with regard to morphology, vigor, litter size, or circadian behavior. We also determined the integration site for both transgenes (see Materials and Methods). *LAP-tTA* was found to be inserted in reverse orientation into the first intron of *Zfp353* (Chromosome 8), more than 100 kilobases (kb) downstream of the first exon and about 200 kb upstream of the second exon. *TRE-Rev-erbα* was found to be inserted in reverse orientation into the first intron of *Semaphorin3ɛ* (Chromosome 5), about 6 kb downstream of the first exon and more than 100 kb upstream of the second exon. We thus consider unlikely that the transgene integrations interfered with circadian clock function.

### The Hepatic Expression of Putative BMAL1 Target Genes in Mice Fed with or without Dox

**Figure 2:**
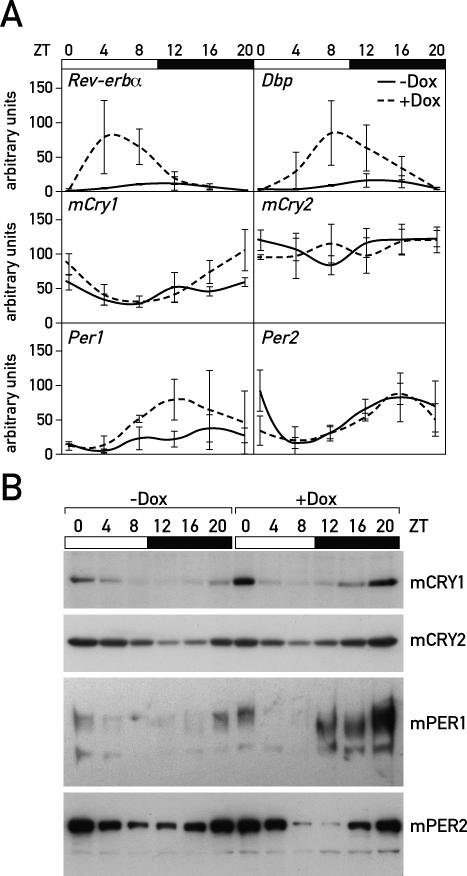
The Expression of Clock and Clock-Controlled Genes Can Be Differentially Affected by HA-REV-ERBα Overexpression (A) TaqMan real-time RT-PCR of cDNA was performed with liver whole-cell RNA for the transcripts of *Dbp,* endogenous *Rev-erbα, mCry1, mCry2, mPer1,* and *mPer2* from untreated *LAP-tTA*/*TRE-Rev-erbα* mice (solid lines; −Dox) and Dox-treated *LAP-tTA*/*TRE-Rev-erbα* mice (dotted lines; +Dox). (B) Western blot analysis of liver nuclear extracts from *LAP-tTA*/*TRE-Rev-erbα* mice that were fed with normal chow (−Dox) or Dox-treated chow (+Dox). In accordance with the temporal mRNA profiles shown in (A) and (B), mCRY1 and mPER1 display reduced levels in untreated mice, whereas mPer2 and mCry2 accumulate to similar levels in nuclei of Dox-treated and untreated animals. The varying mPER1 migration is probably due to oscillating protein phosphorylation and dephosphorylation (see [18]).

As would be expected for BMAL1 target genes, the expression of *mPer1, Dbp,* and endogenous *Rev-erbα* was low in the absence of Dox, when HA-REV-ERBα overexpression attenuated *Bmal1* transcription. Unexpectedly, however, the circadian clock genes *mCry1, mCry2,* and *mPer2* displayed milder expression differences in Dox-treated and untreated animals (Figure 2). Remarkably, the rhythmic expression of *mPer2* mRNA and protein levels was almost unaffected by the down-regulation of *Bmal1* expression. As reported previously [16], mCRY2 oscillated in abundance during the day despite nearly constant *mCry2* mRNA levels. Conceivably, the association of mCRY2 with PER proteins—i.e., mPER2 in the absence of Dox—affected the metabolic stability of mCRY2 in a daytime-dependent manner.

**Figure 3:**
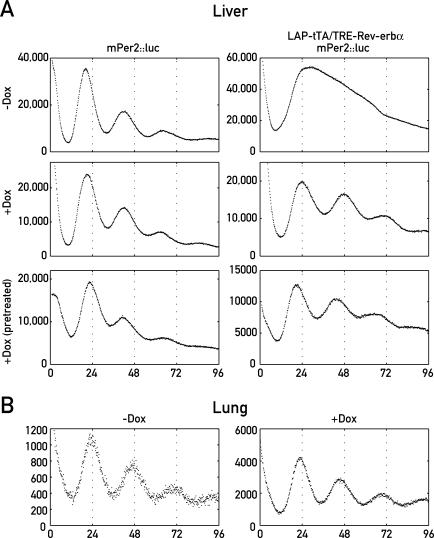
Temporal Luminescence Profiles of Organ Explants from *TRE-Rev-erbα/LAP-tTA*/*mPer2::luc* Triple Transgenic Mice (A) Liver slices from *mPer2::luc* (left) and *LAP-tTA*/*TRE-Rev-erbα*/*mPer2::luc* (right) mice were cultured in luciferin-containing medium in the absence (−Dox) or presence (+Dox) of 10-ng/μl Dox. Luminescence was recorded using photomultiplier tubes. −Dox and +Dox samples are from the same animal; +Dox (pretreated) samples are from mice that have received two intraperitoneal injections of Dox 48 h and 24 h before being sacrificed. (B) Lung explants from *LAP-tTA*/*TRE-Rev-erbα*/*mPer2::luc* mice were cultured as above in the presence or absence of Dox.

The robust circadian expression of *mPer2* in the liver of mice not receiving Dox is in stark contrast to the in situ hybridization experiments with coronal brain sections of *Bmal1*-deficient mice, which indicated that in the absence of BMAL1, *mPer2* mRNA accumulates to insignificant levels throughout the day in SCN neurons [22]. However, it is in keeping with the relatively high constitutive *mPer2* mRNA concentrations observed in the liver of these *Bmal1* knockout mice (J. S. Takahasi, unpublished data), assuming that in liver, *mPer2* transcription depends less on BMAL1 than in the SCN. Nevertheless, our observation could be interpreted in two ways. Either, the residual BMAL1 levels in the liver of animals not treated with Dox were still sufficient to drive *mPer2* transcription, or cyclic *mPer2* expression was governed by oscillating systemic signals in these mice. In order to distinguish between these two scenarios, we wished to monitor temporal *mPer2* expression in cultured liver explants, which obviously do not receive periodic signals from a master pacemaker. To this end, we crossed *LAP-tTA*/*TRE-Rev-erbα* mice with *mPer2::luc* knock-in mice [2], in which a luciferase open reading frame (ORF) was inserted by homologous recombination into the endogenous *mPer2* locus. The mPER2::LUCIFERASE fusion protein encoded by this knock-in allele is fully functional, since it rescues all known rhythm phenotypes of *mPer2* knockout mice [2]. Tissue explants from the *LAP-tTA*/*TRE-Rev-erbα* transgenic mice carrying an *mPer2::luc* fusion allele were placed into culture medium containing luciferin, and bioluminescence was recorded in real time by photomultiplier tubes [2,31]. As shown in Figure 3A (top right panel), liver explants from these mice did not produce circadian luminescence cycles in normal culture medium, suggesting that overexpression of HA-REV-ERBα indeed arrested the hepatocyte clocks. However, when tissue pieces from the same livers were cultured in Dox-containing medium (Figure 3A, right center and bottom panels), circadian luminescence rhythms similar to those observed for explants of *mPer2::luciferase* mice not carrying the *LAP-tTA* and *TRE-Rev-erbα* transgenes (Figure 3A, left panels) could be observed. Interestingly, circadian luminescence cycles recorded from Dox-treated liver explants of *LAP-tTA*/*TRE-Rev-erbα*/*mPer2::luc* mice fed with normal chow (Figure 3A, right center panel), displayed a phase delay of approximately 6 h when compared to those obtained from liver explants of *mPer2::luciferase* mice (Figure 3A, left center panel). This phase delay probably reflected the time period required for the decay of *HA-Rev-erbα* mRNA and protein, and for the consecutive accumulation of BMAL1 to levels compatible with circadian rhythm generation. In keeping with this conjecture, no significant phase differences were observed between luminescence cycles monitored for liver explants from *mPer2::luc* and *LAP-tTA*/*TRE-Rev-erbα*/*mPer2::luc* mice pretreated with Dox by intraperitoneal injections 48 h and 24 h before being sacrificed, (Figure 3A, bottom panels). We have examined liver explants from five mice homozygous (Figure 3A, and unpublished data) and three mice heterozygous (Figure S2A) for the *LAP-tTA*/*TRE*-*Rev-erbα* transgenes, and in all cases, circadian *mPer2::luc* expression strictly depended upon the addition of Dox to the culture medium. As expected, lung explants from either homozygous (Figure 3B) or heterozygous (Figure S2B) *LAP-tTA*/*TRE-Rev-erbα*/*mPer2::luc* mice displayed circadian luminescence rhythms, irrespective of whether or not Dox has been added to the culture medium. Indeed, *TRE-Rev-erbα* transgene expression is not detectable in this tissue by quantitative TaqMan real-time RT-PCR (unpublished data).

Taken together, our observations made with *LAP-tTA*/*TRE-Rev-erbα* mice and tissue explants suggest that in liver, circadian *mPer2* expression can be driven by systemic Zeitgeber cues in the absence of functional hepatocyte clocks as well as by hepatocyte oscillators in the absence of systemic Zeitgeber cues.

### Genome-Wide Mapping of Circadian Transcripts in Liver Cells with Operative or Attenuated Circadian Oscillators

**Figure 4:**
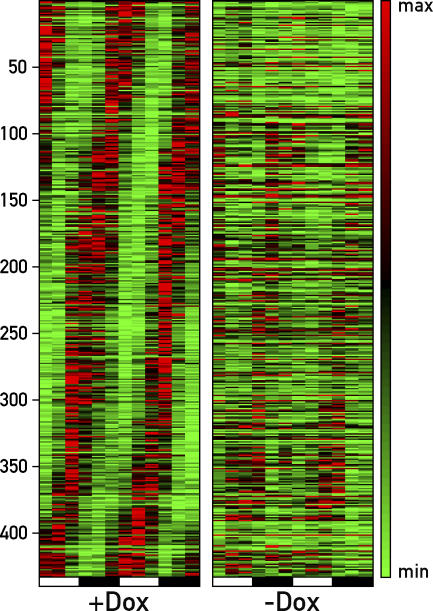
Phase Map of Circadian Transcripts Revealed by Genome-Wide Transcriptome Profiling *LAP-tTA*/*TRE-Rev-erbα* mice fed with Dox-supplemented chow (+Dox) or normal chow (−Dox) were sacrificed at twelve 4-h intervals, and the liver transcriptomes were profiled by Affymetrix oligonucleotide microarray hybridization. Circadian transcripts were retrieved as outlined in Materials and Methods from the 24 datasets, and their temporal expression patterns were aligned according to phase. Note that the circadian accumulation of most transcripts is severely blunted in the livers of untreated mice. The heat scale to the right of the panels represents amplitudes in a linear scale, where green and red represent minimal and maximal expression levels, respectively.

**Figure 5:**
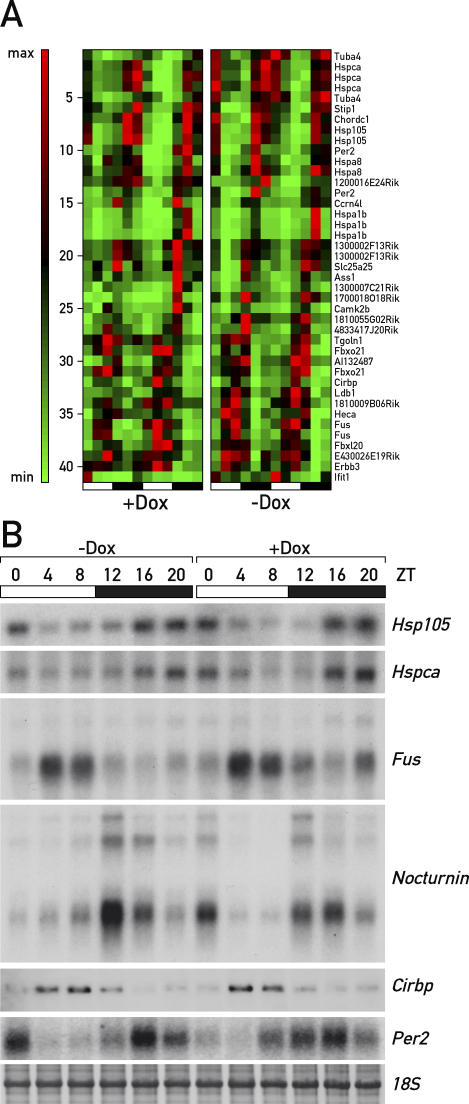
Systemically Driven Circadian Genes Are Unaffected by REV-ERBα Overexpression (A) A subset of transcripts whose circadian accumulation is not significantly affected by the HA-REV-ERBα overexpression is displayed according to the criteria used for (A). We conclude that the circadian rhythms of these genes are driven by systemic timing cues. (B) Northern blot quantification of some systemically driven circadian genes. Some of the results of (A) were validated by Northern blotting. For all six genes tested *(Hsp105, Hspca, Fus, Nocturnin/Ccrn4l, Cirbp,* and *mPer2),* neither amplitude nor phase is affected in a significant manner by REV-ERBα overexpression. The sharpness of the bands for *18S* RNA and *Cirbp* are due to a shorter migration of this particular formaldehyde agarose gel.

To discriminate between oscillator-dependent and -independent circadian gene expression in a genome-wide fashion, we compared the circadian liver transcriptomes of mice fed with or without Dox by Affymetrix (MOUSE 430 2.0) microarray hybridization (for details and data analysis, see Materials and Methods, the microarray data are available from the ArrayExpress repository [http://www.ebi.ac.uk/arrayexpress/] under accession number: E-MEXP-842). This analysis revealed 351 circadian transcripts (represented by 432 feature sets) for Dox-treated animals, including most mRNAs known to fluctuate with a robust daily amplitude (e.g., *mPer1, mPer2, mPer3, mCry1, Rev-erbα, Rev-erbβ, Bmal1, Clock, Dbp, Tef, Nocturnin, Rorγ, E4bp4, Cyp7a1,* or *Alas1*)*.* In keeping with previous studies [23–27], many cyclically expressed genes are involved in various aspects of liver physiology such as xenobiotic detoxification (e.g., *P450 oxidoreductase, Por, Cyp2b9, Cyp2b10, Cyp2g1,* and *Fmo5*), carbohydrate and energy metabolism (e.g., *Gk,* and *Pepck*), or lipid and sterol homeostasis (e.g., *Elovl3, Insig2, Lipin1,* and *Cyp7a1*). Importantly, the cyclic expression of most rhythmically active genes appeared to depend on an intact hepatocyte oscillator, as the amplitude of circadian accumulation was greatly affected in animals not receiving Dox-supplemented food (Figure 4). Nevertheless, using the algorithms described in Materials and Methods, we identified 31 different transcripts (represented by 41 feature sets), whose circadian accumulation was not affected by the Dox treatment. These are listed in the phase maps of Figure 5A (compare left and right panels), and the circadian expression of some of these genes in the presence and absence of Dox has been validated by Northern blot hybridization (Figure 5B). As expected on the basis of the results displayed in Figure 2, *mPer2* mRNA was included among the transcripts whose cyclic accumulation was controlled by systemic cues. Other genes whose transcripts accumulate with phase angles similar to that of *mPer2* mRNA were the heat-shock protein genes *Hspca* (encoding HSP90), *Hspa8* (encoding HSP70 isoform 8), *Hspa1b* (encoding HSP70 isoform 1A), *Hsp105* (encoding HSP105), and *Stip1* (encoding Stress-Induced Phoshoprotein 1, also known as Hsp70/Hsp90 organizing protein), and a tubulin gene *(Tuba4)*. These genes were expressed in phase with *mPer2,* suggesting that their cyclic transcription was perhaps governed by similar systemic timing cues. *Nocturnin (Ccrn4l), Fus, Chordc1,* and *Cirbp* were additional genes whose circadian expression appeared to be system driven. However, the transcripts issued by these genes belonged to different phase clusters (Figure 5A and 5B), and their synthesis must thus have been regulated by mechanisms different from those governing rhythmic *Hsp* and/or *mPer2* transcription. Particularly interesting was the diurnal expression of heat-shock protein genes and *Cirbp,* a gene encoding a cold-induced RNA-binding protein. While heat-shock protein mRNAs reached zenith levels at Zeitgeber times when body temperature was maximal, *Cirbp* mRNA levels peaked at Zeitgeber times when body temperature was minimal [5,32]. Hence temperature cycles oscillating by only a few degrees (35 °C to 38 °C) appeared to be translated into antiphasic *Hsp* and *Cirbp* expression cycles.

We also considered the possibility that some systemically regulated liver genes could display mRNA accumulation cycles with higher amplitudes in the absence of functional hepatocyte oscillators. For example, the accumulation of liver transcripts whose synthesis is influenced by local oscillators and systemic cues in an antiphasic manner may only be circadian in mice not containing hepatocyte clocks. However, our failure to identify such transcripts did not support such a regulatory mode (see Figure S3A and S3B, and corresponding figure legends), we thus feel that few if any genes produce robust daily mRNA accumulation cycles only in the absence of functional hepatocyte clocks.

### Temperature-Dependence of *mPer2* Expression

**Figure 6:**
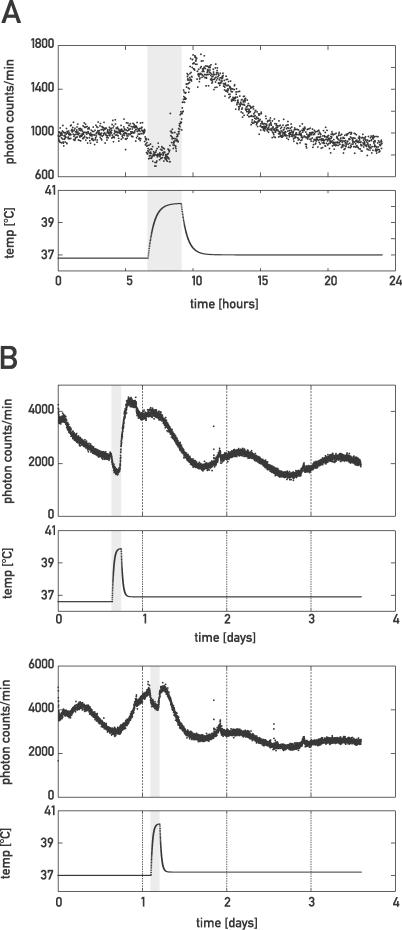
Heat-Shock Induction of *mPer2* (A) Liver explants of *LAP-tTA*/*TRE-Rev-erbα*/*mPer2::luc* mice were cultured as in Figure 3 and subjected to heat shock (150 min at ∼40 °C) using homemade culture-dish heating devices, and luminescence was recorded as in Figure 3. Temperature plots are extrapolated from periodic temperature measurement. The time window during which organ cultures were exposed to an elevated temperature is depicted by a grey box. (B) Lung explants were subjected to heat shock as in (A) at two different circadian times.

The expression of *mPer2* and *Hsp* appears to be similar with respect to systemic regulation. We thus suspected that a common regulator might influence the transcription of these genes. Since *Hsp* transcription is governed primarily by heat-shock transcription factors (HSF) [33], we wondered whether *mPer2* transcription was also inducible by elevated temperature. In order to examine this conjecture, we incubated cultured organ explants from *LAP-tTA*/*TRE-Rev-erbα*/*mPer2::luc* mice during 150 min at 40 °C (Figure 6) and recorded bioluminescence in real time. Although luciferase activity was somewhat decreased during the heat shock itself, presumably due to a general inhibition of translation [34], a subsequent strong increase in luciferase activity was observed in both liver and lung. Liver explants cultured in the absence of Dox showed a consistent 2-fold enhancement of luciferase activity, suggesting that the heat-dependent regulation of *mPer2* did not require a circadian clock (Figure 6A). Lung explants, in which circadian oscillators are operative under these conditions (see Figures 3B and S2B), displayed a phase-specific induction of temperature-induced luciferase activity (Figure 6B). Thus, when the heat shock was performed at a circadian time at which luciferase activity was minimal, a strong induction was observed. On the other hand, a heat shock performed at a circadian time when luciferase activity was maximal did not result in a noteworthy increase in luciferase activity. Taken together, these results indicate that *mPer2* is heat inducible and that the strength of this induction is gated by circadian time.

The minimal HSF binding sites (heat-shock elements [HSEs]) consist of two or more inverted or everted repeats of the pentameric sequence 5′-NGAAN-3′ (where N can be any nucleotide). Taking the two complementary DNA strands into consideration, the statistical frequency of HSEs is approximately 1/2,000 in random DNA, and it is thus impossible to identify functional HSEs solely by sequence inspection. Nevertheless, known functional HSEs are located within 5′-flanking regions of heat-shock protein genes [35], and the sequence analysis of *mPer2* revealed a cluster of five HSEs within the 1,700 base pairs (bp) located upstream of the transcription initiation site (Figure S4). Of note, one of these elements (centered around −1,630) lies within a 22-bp sequence block that is 100% identical in mouse, rat, human, and dog (Figure S4). Whether this or any other HSEs displayed in Figure S4 are involved in the temperature-regulation of *mPer2* will have to be examined by site-directed mutagenesis and chromatin immunoprecipitation experiments.

## Discussion

We generated a mouse model system in which hepatocyte circadian oscillators can be attenuated in a conditional fashion. The system is based on the tetracycline-dependent, liver-specific overexpression of the nuclear orphan receptor REV-Erbα, a potent repressor of the essential clock gene *Bmal1*. Thus, when the tetracycline analog doxycycline was omitted from the food, REV-ERBα accumulated to high levels throughout the day and thereby inhibited *Bmal1* transcription constitutively in *LAP-tTA*/*TRE-Rev-erbα* mice. As a consequence, the expression of obligatory *Bmal1* target genes was decreased to a level that no longer supports local oscillator function. When doxycycline was added to the food, the *Rev-erbα* transgene was silenced, and hepatocyte oscillator function was reestablished in *LAP-tTA*/*TRE-Rev-erbα* mice.

**Figure 7:**
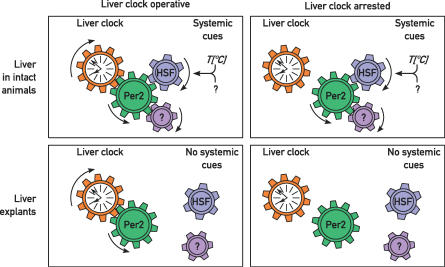
Model for the Synchronization of Liver Oscillators In the intact animal, the phase of circadian mPer2 cycles is dictated by systemic Zeitgeber cues such as temperature or chemical cues influencing HSF activity (see text). Since mPER2 is also an integral part of the clockwork circuitry, this protein might confer the phase of systemic Zeitgebers to the local oscillator. If the oscillator is inactivated (e.g., by the repression of *Bmal1*), mPER2 is still expressed in a circadian manner in the intact animal. Under free-running conditions (i.e., in liver explants cultured in vitro), rhythmic *mPer2* expression persists, but with the phase and period imposed by the local oscillators. However, when this oscillator is arrested, the expression of *mPer2* and probably that of all clock and clock-controlled genes becomes arrhythmic.

By using this novel mouse model, we were able to discriminate between genes whose cyclic expression is driven either by local hepatocyte oscillators or by systemic circadian cues that are controlled directly or indirectly by the SCN. The transcription of liver genes whose expression displayed daily oscillations in *LAP-tTA*/*TRE-Rev-erbα* mice despite arrested hepatocyte clocks are likely under the control of physical and/or chemical cues whose systemic rhythms are driven by the central SCN pacemaker. Such systemically regulated genes are expected to include genes involved in the synchronization of hepatocyte clocks. Genome-wide profiling of the liver circadian transcriptome of *LAP-tTA*/*TRE-Rev-erbα* mice fed with Dox-supplemented chow revealed about 350 transcripts with robust circadian accumulation. Less than 10% of these transcripts displayed rhythmic accumulation with high amplitude and magnitude in mice fed with normal chow, suggesting that the cyclic transcription of most circadian genes is influenced by local oscillators. We cannot formally exclude that the cyclic expression of some of the genes resilient to HA-REV-ERBα overexpression was driven by a second, yet unknown and BMAL1-independent oscillator. However, this hypothesis clearly did not apply to *mPer2,* since mPER2::LUC expression ceased to be rhythmic in liver explants not treated with Dox. We thus consider it more likely that the rhythmic expression of genes in the absence of Dox was governed by systemic cues, which were directly or indirectly controlled by the master pacemaker in the SCN. As illustrated in Figure 7, the system-driven circadian expression of *mPer2* is of particular interest with regard to the entrainment of the peripheral oscillators by SCN-borne timing cues. The oscillatory mechanism that is at the center of the circadian clock is thought to involve a negative feedback of CRYs and PERs on their own transcription. An externally driven *mPer2* transcription cycle would thus gate the phase of the peripheral clock to that of the systemic signals. Indeed, the system-driven expression of *mPer2* provides a direct link between circadian systemic signals and the phase of peripheral oscillators. Although the molecular mechanisms responsible for system-driven *mPer2* transcription remain to be identified, the observation that many heat-shock protein genes were found to be expressed in phase with *mPer2* suggests that the cyclic transcription of *mPer2* and *Hsp* genes shares certain regulatory mechanisms. Of note, real-time bioluminescence recordings of mPer2::luc-expressing liver and lung explants exposed to a heat shock showed that *mPer2* transcription can indeed be influenced by temperature. Moreover, the 5′-flanking region of *mPer2* harbors five heat-shock response elements (HSEs) within 1,700 bp, of which one is 100% identical in mouse, rat, man, and dog. The identification of the physiologically relevant HSEs within the *mPer2* gene will be particularly important, since the activity of HSF1 can also be influenced by chemical cues (e.g., oxidants) [33]. As feeding cycles are the most dominant Zeitgebers for peripheral clocks thus far identified, it is tempting to speculate that HSF1 senses rhythmic metabolism and thereby synchronizes peripheral clocks by gating *mPer2* expression.

Similar to certain nuclear hormone receptors, HSF1 forms functionally inert cytoplasmic complexes with chaperones and co-chaperones in the absence of activating cues [36]. Upon exposure to elevated temperature, oxidative stress, heavy metals, or endobiotic substances (e.g., arachidonic acid), HSF1 gets activated in multiple consecutive steps [37]. These comprise: release from chaperones and co-chaperones, trimerization via an unmasked coiled-coil domain, binding to its cognate DNA sequences in regulatory regions of target genes, and stimulation of the transactivation potential via the calcium/calmodulin-dependent kinase II (CAMKII)-mediated phosphorylation of a serine residue within the HSF1 regulatory domain [38]. Of note, *CamkIIb* mRNA is among the transcripts whose diurnal accumulation is governed by systemic cues and whose phase is in keeping with a role of CAMKIIb in the circadian activation of HSF1 (see Figure 5A). In addition, CAMKIIb might also participate in the synchronization of peripheral clocks by a more direct mechanism. Thus, in fruit fly cells, CAMKII phosphorylates CLK, the *Drosophila* ortholog of CLOCK, and in cotransfection experiments. this enhances the stimulation of CLK-CYC target genes [39].

*Nocturnin* and *Cirbp* are two systemically driven genes encoding proteins potentially involved in mRNA metabolism and/or activity. Nocturnin, the vertebrate homolog of yeast CCR4, is an mRNA deadenylase [40] with rhythmic expression in many mouse tissues [41]. As both mRNA stability and translation efficiency can depend on poly(A) length, Nocturnin could influence the rhythmic accumulation of circadian proteins by post-transcriptional mechanisms. Likewise, CIRBP, a nuclear, ubiquitously expressed RNA-binding protein [42], could affect the cyclic accumulation or translation of target mRNAs in a temperature-dependent fashion, as diurnal *Cirbp* expression correlates negatively with body temperature rhythms. In addition, CIRBP has been demonstrated to activate the extracellular signal-regulated kinase (ERK) pathway in NIH3T3 fibroblasts [43]. Similar to CAMKII, *Drosophila* ERK2 can phosphorylate CLK and thereby increase the transactivation potential of this transcription factor. *Cirbp* mRNA and protein levels have previously been found to oscillate in brain, but not in liver [32]. However, in the latter tissue, the accumulation of *Cirbp* transcripts has been determined only for two time points, and the cyclic accumulation of *Cirbp* mRNA in liver (see Figure 5B) may thus have escaped this analysis. The similarly high amplitude of diurnal *Cirbp* mRNA accumulation in brain [32] and liver (this study) is somewhat surprising. In fact, most brain areas display much shallower accumulation cycles for clock and clock-controlled mRNAs than the liver. For example, circadian *mPer1* and *Dbp* mRNA levels oscillate about 13-fold and 100-fold, respectively, in liver, but only about 1.4-fold and 2-fold, respectively, in brain [44]. The low-amplitude rhythms in the latter tissue may be the consequence of an incomplete phase entrainment of local oscillators in brain neurons by the SCN, perhaps because the transport of chemical timing cues across the blood-brain barrier is inefficient. The high amplitude of *Cirbp* expression in the brain may thus be caused by daily temperature fluctuations, which have similar amplitudes in the brain and peripheral organs [45].

In conclusion, we have established a transgenic mouse model that allowed us to study rhythmic liver gene expression genome-wide in the presence and absence of functional hepatocyte oscillators. The identification of genes whose amplitude and phase are nearly identical under these two conditions revealed possible mechanisms by which peripheral oscillators could be entrained. The observation that in liver the circadian expression of *mPer2* can be governed by both systemic cues and hepatocyte oscillators provides a plausible mechanism for the phase entrainment of molecular oscillators in peripheral tissues. Strikingly, heat-induced and cold-induced genes were also identified among the genes whose rhythmic expression is driven by systemic cues. Of note, body temperature rhythms have previously been shown to contribute to the phase entrainment of peripheral clocks [8], and it is thus tempting to speculate that the molecular mechanism governing temperature-dependent *Hsp* and/or *Cirbp* expression are involved in this process. We feel confident that the in-depth analysis of *cis*-acting regulatory elements and transcription factors participating in the systemic control of circadian gene transcription will provide valuable information on the phase-entrainment pathways operative in peripheral tissues.

## Materials and Methods

### Generation of *TRE-Rev-erbα* transgenic mice.

*TRE-Rev-erbα* mice were generated by pronuclear injection as described in [46]. A cDNA containing the full-length REV-ERBα coding sequence was obtained from F. Damiola. This cDNA contains the first 134 bp of the mouse cDNA (up to the BamHI site) preceded by two HA tags and followed by the remaining of the rat REV-ERBα sequence (F. Damiola and U. Schibler, unpublished data). The cDNA sequence was then PCR amplified using the primers “Bcl1-HA-Revα” and “Bcl1-downstream-Revα” (see Table S1). The PCR product was cut with BclI and cloned into the BamHI site of the pTRE-2 plasmid (ClonTech, Mountain View, California, United States). This new plasmid was then cleaved with XhoI and AseI, and the resulting 3,645-bp fragment encompassing the seven TREs, the minimal CMV promoter, and the HA-tagged REV-ERBα ORF followed by the rabbit β-globin 3′UTR, was used for microinjection into the pronuclei of mouse zygotes. The transgenic mouse line used in this study was selected among 21 lines obtained from 21 different founder mice for its high and strictly Dox-dependent expression of the *TRE-Rev-erbα* transgene (as assessed by TaqMan real-time RT-PCR).

### Dox treatment.

Dox-containing food pellets were produced as follows: powdered mouse chow (Provimi Kliba, Kaiseraugst, Switzerland) was mixed with an equal weight of water containing 3-g/l Dox (Ufamed, Sursee, Switzerland). The suspension was allowed to stand for a few hours in order to saturate the powder with the Dox solution. Small pellets were then formed, and the water was removed by vacuum lyophilization. Mice were fed with these food pellets for at least 1 wk before they were sacrificed for the analysis of RNA and protein.

### Determination of the transgene insertion sites.

We determined the chromosomal insertion site for the *TRE-Rev-erbα* and *LAP-tTA* transgenes in order to facilitate the genotyping analysis of transgenic mice by PCR experiments. To this end, transgenic genomic DNA was digested with a frequently cutting restriction enzyme that cleaves the transgene at defined sites (NlaIII for *TRE-Rev-erbα* and Sau3AI for *LAP-tTA*). After heat inactivation of the restriction enzyme, the DNA was diluted to a concentration of 2 ng/μl and ligated with T4 DNA ligase in order to circularize the DNA restriction fragments. These DNA fragments were then precipitated and re-linearized with an infrequently cutting restriction enzyme (SacI in both cases) that cuts the transgene between the restriction sites previously used for the production of circularized DNA fragments (composed of transgene and flanking genomic sequences). The DNA was then used for PCR amplification with primers “TRE-fwd” and “TRE-rev” for *TRE-Rev-erbα* and “pLAP-fwd” and “pLAP-rev” for *LAP-tTA* (see Table S1). The resulting PCR products were sequenced and the insertion sites determined.

*Lap-tTA* genotyping was performed by PCR using the primers “LAPtTAtg-fwd” for the transgenic allele, “LAP-tTAwt-fwd2” for the wild-type (WT) allele, and “LAPtTA-rev2” as common reverse primer (see Table S1). The resulting PCR products encompass 302 bp for the WT allele and 360 bp for the transgenic allele. Genotyping of *TRE-Rev-erbα* was performed by PCR using the primers “twdTRE” for the transgenic allele, ‘TREvalphaWT2” for the WT allele, and ‘TREvalpha-rev2” as common reverse primer (see Table S1). The resulting PCR products span 351 bp for the WT allele and 560 for the transgenic allele. All experiments shown in the main text of the paper were conducted using double homozygous mice for both *Lap-tTA* and *TRE-Rev-erbα* in order to maximize the expression of the transgene, whereas all mice used for in vitro liver explants were heterozygous for the *mPER2::LUC* allele. The experiments presented in Figure S2 were performed with *Lap-tTA*/*TRE-Rev-erbα*/*mPER2::LUC* triple heterozygous mice for reasons outlined in the legend to this figure.

### RNA analysis.

RNA expression levels were determined using whole-cell RNA essentially as described in [16]. Liver whole-cell RNA was extracted according to reference [47], and Northern blot experiments were performed using 5 μg of whole-cell RNA and hybridization to radiolabeled DNA probes according to the Church protocol [48]. *Bmal1* and *mPer2* specific probes were generated using sequences encompassing the entire open reading frames as templates. For the Northern blot experiments displayed in Figure 5B, hybridization probes were generated from cloned PCR products encompassing the following sequences: bp 1,144 to 1,946 of *Nocturnin,* bp 429 to 1,583 of *Fus,* bp 2,325 to 2,991 of *Hsp105/110,* bp 1,032 to 1,784 of *Hspca/Hsp90,* and bp 552 to 1,139 of *Cirbp*. Real-time TaqMan RT-PCR was performed as described [16]. The primers and probes used in this study are all listed in Table S1. A primer-probe set for the Tata binding protein (TBP) transcript was used for normalization. The same TaqMan probe was used for mouse and rat *Rev-erbα,* because the mouse and rat sequences are identical in the region encompassing this DNA segment.

### Western blotting.

Nuclear extracts were prepared by the NUN procedure as described [49], and Western blotting was performed according to standard protocols using affinity-purified rabbit polyclonal antibodies. αCry1, αCry2, αPer1, αPer2, αBmal1, and αREV-ERBα antibodies were kindly provided by S. Brown and J. Ripperger.

### Bioluminescence analysis in liver explants.

Bioluminescence measurements of liver slices were performed essentially as described [2]. Mice were sacrificed by decapitation. The inferior vena cava was cut, and ice-cold Hank's Balanced Salt Solution (HBSS, Sigma Cat no. H1641; St. Louis, Missouri, United States) was perfused through the spleen in order to remove blood and refrigerate the liver. Tissue pieces were removed, placed into ice-cold HBSS, and sliced into smaller fragments (volume approximately 10 mm^3^). These tissue pieces were then placed on glass fiber filters in 35-mm tissue culture dishes containing 1.2–1.5 ml of HEPES-buffered phenol red-free DMEM (GIBCO Cat no. 1741; San Diego, California, United States) supplemented with 5% fetal calf serum, 2 mM glutamine, 100-U/ml penicillin,100-μg/ml streptomycin, and 0.1 mM luciferin. Only distal edges of the liver lobes were used, since they gave more reproducible and persistent cycles, perhaps owing to their favorable surface/volume ratio. When relevant, Dox was added to a final concentration of 10 μg/ml. Cultures were maintained at 37 °C in a light-tight incubator, and bioluminescence was monitored continuously using Hamamatsu photomultiplier tubes (PMT; Hamamatsu, Hamamatsu City, Japan) [31]. Photon counts were integrated over 1-min intervals. Dox pretreatment of animals (Figure 3) consisted of two intraperitoneal injections of 2-mg Dox in PBS. Temperature variations in tissue explant cultures were generated using homemade programmable heating chambers.

### Microarray hybridization.

Thirty-six male mice double homozygous for the two transgenes fed with normal chow and an equal number of mice fed with Dox-supplemented chow were maintained on an LD12:12 light cycle and were used for these experiments. Six animals each for Dox-treated and untreated mice were sacrificed at ZT00, ZT04, ZT08, ZT12, ZT16, and ZT20, and whole-cell liver RNA was extracted from each animal. RNA pools of three male animals were assembled by mixing equal amounts of RNA. This resulted in 12 temporally staged RNA pools representing two entire days. A total of 5 μg of pooled RNA was used for the synthesis of biotinylated cRNA; 8.75 μg of biotinylated cRNA was then hybridized to 24 mouse Affymetrix 430 2.0 chips containing 45,000 feature sets and representing 39,000 genes, using standard Affymetrix protocols. The chips were washed and scanned, and the fluorescence signals were analyzed with the RMAexpress software using Robust Multi-array Analysis (RMA) [50].

The data thus obtained were used for a Fourier transform analysis, and the ratio of the F_24_ spectral power to the sum of the other Fourier components (i.e., ∞, 48, 12, 9.6, and 8 h) was calculated for each feature set [51]. The time points were then permuted 50,000 times, and for each of the permutations, the Fourier transform was calculated together with the ratio of the F_24_ Fourier component to the other components [52]. Expression data were fitted to a cosine curve. Temporal mRNA accumulation was considered as circadian if its amplitude was higher than 2-fold and if the ratio of the unscrambled Fourier transform was within the top 5% of the scrambled ratios. In order to find circadian genes unaffected by Dox treatment, we calculated a divergence *(D)* coefficient as follows:

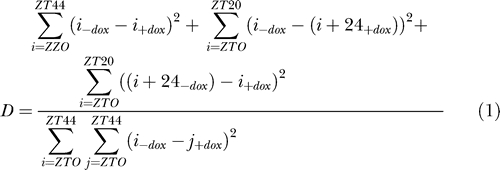
where ZT0:ZT44 represents the hybridization signal for the specified time points. We did not apply any filter for “presence” or “absence” flags (as determined by the MAS Affymetrix software), since 95% of the hybridization signals obtained for the 432 feature sets that we qualified as circadian by Fourier transform analysis scored as “present” in more than 12 experiments out of 24 by the MAS software. By comparison, only 35% of total (circadian and non-circadian) features sets were considered as present in at least 12 experiments.

## Supporting Information

Figure S1 Quantification of *Bmal1* Protein Levels in *TRE-Rev-erbα/LAP-tTA* Transgenic Mice (71 KB PDF)

Figure S2 Temporal Luminescence Profiles of Liver and Lung Explants from Triple Heterozygous *TRE-Rev-erbα/LAP-tTA/mPer2::luc* Triple Transgenic Mice (2.8 MB PDF)

Figure S3 Detection of Transcripts Displaying Circadian Accumulation Profiles in Mice Fed with Normal Chow (−Dox) (192 KB PDF)

Figure S4 Identification of Potential HSF Binding Sites in the *mPer2* Gene (302 KB PDF)

Table S1 PCR Primers and Probes Used in This Study (47 KB PDF)

### Accession Numbers

The GenBank (http://www.ncbi.nlm.nih.gov/Genbank) accession numbers for the genes and gene products discussed in this paper are *Cirbp* (NM_007705), *Fus* (NM_139149), *Hsp105/110* (NM_013559), *Hspca/Hsp90* (NM_010480), and *Nocturnin* (NM_009834).

The ArrayExpress repository (http://www.ebi.ac.uk/arrayexpress) accession number for the microarray data is E-MEXP-842.

## Abbreviations

bp: base pair
CAMKII: calcium/calmodulin-dependent kinase II
Dox: doxycycline
HSE.: Heat-shock element
HSF: heat-shock transcription factor
kb: kilobase
SCN: suprachiasmatic nucleus
TRE: tetracycline-responsive element
tTA: tetracycline-dependent transactivator
WT: wild-type
