# Supplementary material for: System-Driven and Oscillator-Dependent Circadian Transcription in Mice with a Conditionally Active Liver Clock
Source: PLoS Biol. 2007 Jan 30;5(2):e34. doi: 10.1371/journal.pbio.0050034 (PMC1783671; doi:10.1371/journal.pbio.0050034)
Supplement: Supplementary file 1 [file pbio.0050034.sg001.pdf]

# Figure S1

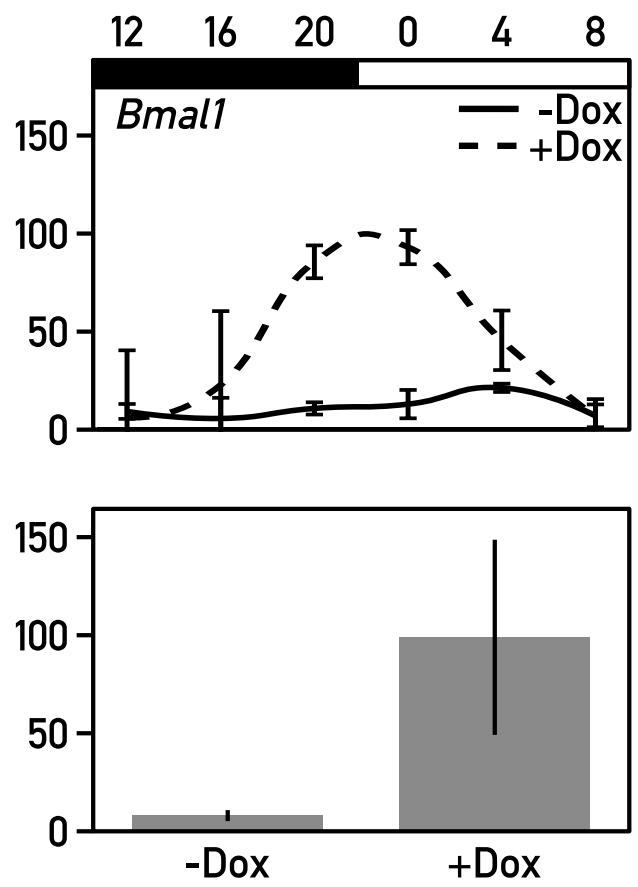

**Figure S1:** Quantification of *Bmal1* protein levels in *TRE-Rev-erbα/LAP-tTA* transgenic mice. Upper panel: RT-TaqMan qPCR was performed for *Bmal1* mRNA employing the cDNAs used for the data presented in figure 2. Integration of the qPCR signals revealed a decrease of four fold for *Bmal1* mRNA in mice overexpressing HA-REV-ERBα.

Lower panel: densitometry analysis of the Western signals shown in Figure 1C. Since the BMAL1 protein does not show a significant circadian variation in abundance, all 6 time points of the –Dox (left) and +Dox (right) have been averaged and the standard deviation is represented by the error bar. This analysis suggests tha BMAL1 protein expression is downregulated about 12 fold by HA-REV-ERBα overexpression.
