# Supplementary material for: System-Driven and Oscillator-Dependent Circadian Transcription in Mice with a Conditionally Active Liver Clock
Source: PLoS Biol. 2007 Jan 30;5(2):e34. doi: 10.1371/journal.pbio.0050034 (PMC1783671; doi:10.1371/journal.pbio.0050034)
Supplement: Supplementary file 2 [file pbio.0050034.sg002.pdf]

Figure S2

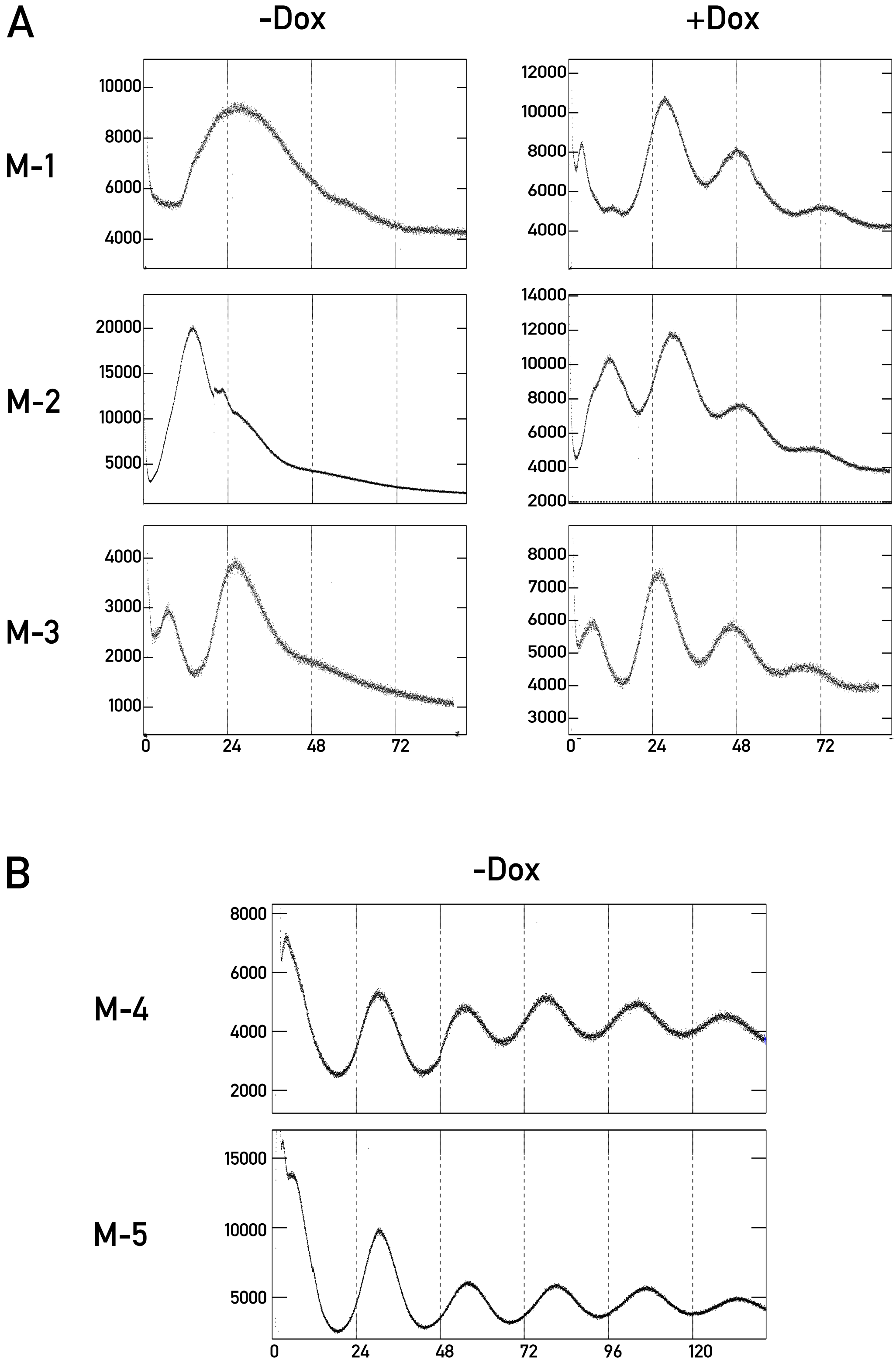
A
-Dox
+Dox
M-1
10000
8000
6000
4000
12000
10000
8000
6000
4000
M-2
20000
15000
10000
5000
14000
12000
10000
8000
6000
4000
2000
M-3
4000
3000
2000
1000
8000
7000
6000
5000
4000
3000
0
24
48
72
0
24
48
72
B
-Dox
M-4
8000
6000
4000
2000
M-5
15000
10000
5000
0
24
48
72
96
120

Figure S2: Temporal luminescence profiles of liver and lung explants from triple heterozygous *TRE-Rev-erbα/LAP-tTA/mPer2::luc* triple transgenic mice.

A) Liver slices were cultured in luciferin-containing medium in the absence (-Dox) or presence of 10 μg/ml Dox (+Dox). Luminescence was recorded using photomultiplier tubes. Slices from the liver of three different mouse individuals (M-1, M-2, M-3) are presented, and the results obtained for untreated and Dox-treated explants from these livers are shown in the left and right panels, respectively. Circadian luminescence cycles are only observed with the Dox-treated explants. The data obtained with mice heterozygous for the *TRE-Rev-erbα* and *LAP-tTA* transgenes are basically the same as those shown in Figure 3 obtained with mice homozygous for these transgenes. Hence, even in liver explants from heterozygous animals the downregulation of *Bmal1* transcription in the absence of Dox is sufficient to abolish circadian mPer2::luc expression.

B) Luminescence cycles obtained with lung explants from two triple heterozygous individuals (M-4, M-5) cultured in the absence of Dox are shown. Since lung cells do not express the *LAP-tTA* transgene, circadian *mPer2::luc* expression persists in the absence of Dox. Note that lung slices yield more robust luminescence cycles and survive longer in culture than liver slices. This probably reflects the high surface/volume ratio of pulmonary tissue, assuring sufficient oxygen and nutrient supply to most cells of the explant during extended time periods.
