# Supplementary material for: System-Driven and Oscillator-Dependent Circadian Transcription in Mice with a Conditionally Active Liver Clock
Source: PLoS Biol. 2007 Jan 30;5(2):e34. doi: 10.1371/journal.pbio.0050034 (PMC1783671; doi:10.1371/journal.pbio.0050034)
Supplement: Supplementary file 3 [file pbio.0050034.sg003.pdf]

Figure S3

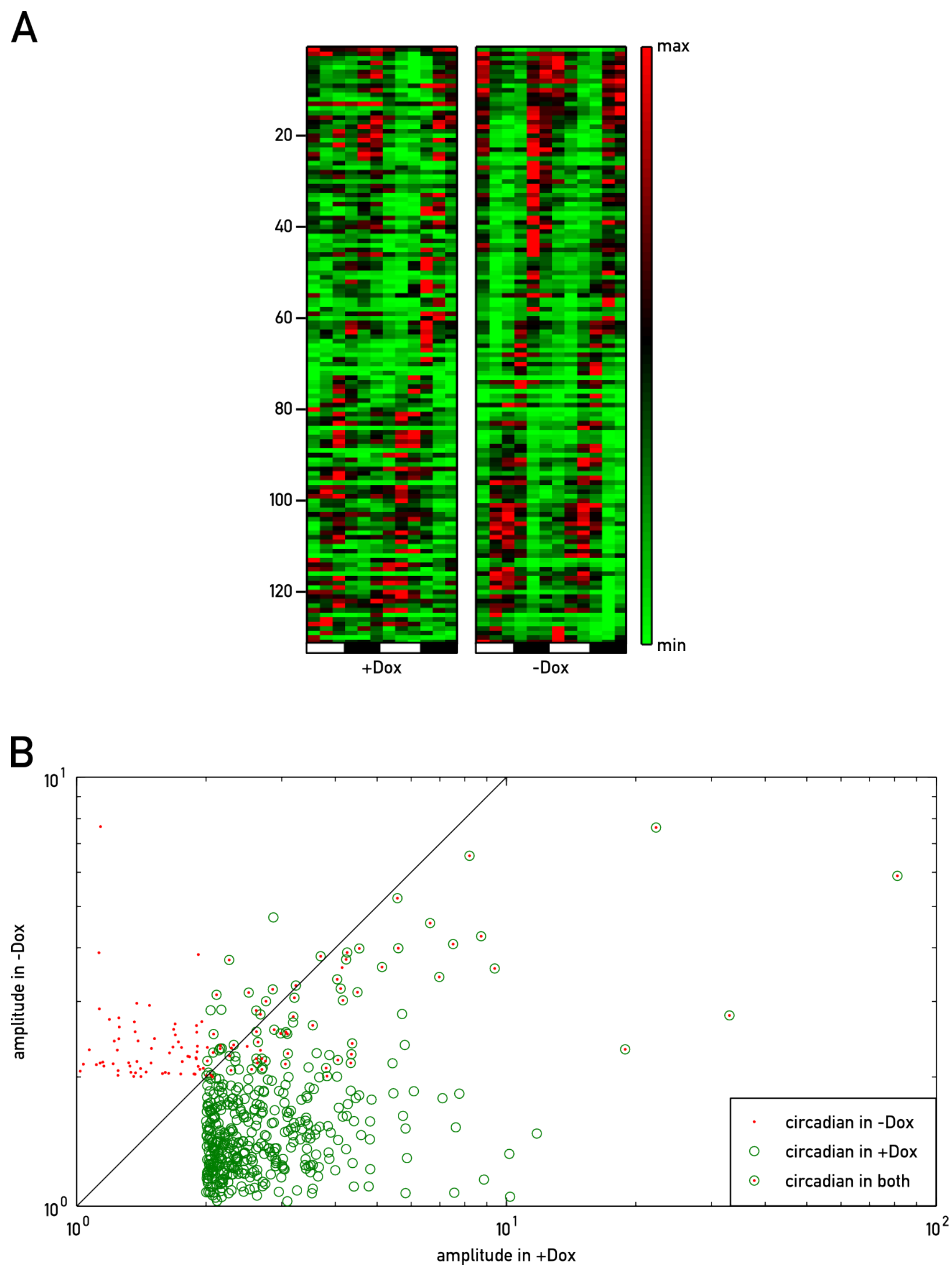
A
20
40
60
80
100
120
max
min
+Dox
-Dox
B
amplitude in -Dox
amplitude in +Dox
10^0
10^1
10^2
circadian in -Dox
circadian in +Dox
circadian in both

Figure S3: Detection of transcripts displaying circadian accumulation profiles in mice fed with normal chow (-Dox).

A) Phase map of the 131 hepatic feature sets displaying circadian accumulation in *TRE-Rev-erbα /LAP-tTA* mice fed with normal chow. Transcripts that met the criteria for circadian accumulation in –Dox conditions according to the algorithms used (see Materials and Methods) were retrieved and their temporal accumulation patterns were aligned according to phase as in Figure 4. Note that most of them display also a circadian accumulation profile in +Dox conditions, indicating that the expression of these genes was either unaffected by the Dox treatment (see Figure 5) or downregulated, but still circadian. The circadian hybridization signals for these may have been contributed by non-hepatocyte cell types.

B) Plot of the amplitudes of circadian mRNA accumulation cycles in mice fed with normal chow (–Dox) or Dox-supplemented chow (+Dox). The amplitude (as defined by the ratio of the maximum to minimum of the best-fit cosine function, see Material and Methods) of the transcripts displaying a circadian accumulation in –Dox conditions (red dots) and +Dox conditions (green open circles) was plotted. The genes that are considered as circadian in both conditions are depicted by both marks. Abcissa: Amplitude of mRNA accumulation cycles obtained from mice fed with normal chow (-Dox). Ordinate: Amplitude of mRNA accumulation cycles obtained from mice fed with Dox-supplemented chow (+Dox). The vast majority of genes showing a high amplitude in –Dox (e.g. more than three fold) are also circadian in +Dox. Note that the opposite is not true.

From these data we conclude that the transcripts considered as circadian in the –Dox conditions are part of one of following three categories: (1) circadian transcripts whose accumulation is unaffected by Dox treatment, because their rhythmic transcription is regulated by systemic cues (e.g. *mPer2*), (2) circadian transcripts, such as *Dbp* mRNA, whose accumulation displays low-magnitude, but detectable circadian amplitudes, presumably owing to their expression in non-hepatocyte cell types (e.g. Kupffer cells, endothelial cells, bile duct cells, which together constitute about 10% of the liver mass), and (3) circadian transcripts whose transcription is regulated in an additive or synergistic fashion by systemic cues and oscillator components in hepatocytes. Note that

the transcripts belonging to these categories are circadian in untreated and Dox-treated animals. Finally, transcripts that display circadian accumulation in untreated animals but not in Dox-treated mice show amplitudes and/or Fourier p values that are close to the threshold values imposed by our algorithms. Hence, we consider likely that most if not all of the cyclic profiles contributed by such transcripts may represent either false positives under –Dox conditions or false negatives under +Dox conditions.
