# Supplementary material for: System-Driven and Oscillator-Dependent Circadian Transcription in Mice with a Conditionally Active Liver Clock
Source: PLoS Biol. 2007 Jan 30;5(2):e34. doi: 10.1371/journal.pbio.0050034 (PMC1783671; doi:10.1371/journal.pbio.0050034)
Supplement: Supplementary file 4 [file pbio.0050034.sg004.pdf]

Figure S4

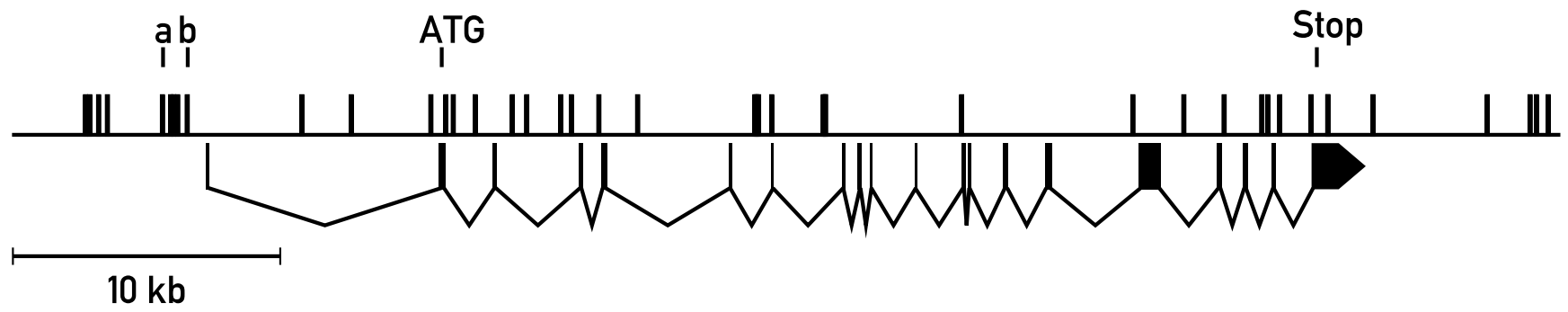

a

```
mouse GTGGATGAG----CTGTGTACTCTTGTTTCCAGAACAATGTAGC-CACCATTGCCGTCAATGTAAGCGAGGAAACAAAAGGCCCTTTGGGTGT
  rat GTGGATGAG----CTGTGTACTCTTGTTTCCAGAACAATGCAGC-CACCATTGACGTCAATGAAAGTGAGGAAACAAAAGGCCCTTTGGGTGT
human GTGGATGAGCTGTCTGTTTACTCTTGTTTCCAGAACAATGGAGCTCCCCATTGACGTCAATGGGAGGGAAGAAACAAAAGAGCCTTTGGGTGT
  dog GTGGATGAGCTGTCTGTTTACTCTTGTTTCCAGAACAATGGAGCTTACCATTGACGTCAATGTACATGAAGGAACAAAAG-GCCTTTGGGTGT
```

b

```
mouse TTCTTTCCTTTGTATCTCTGCACGGCAATTATGACCTT-ATTTCCTGAATCAACACTAACTAGCAACACGCAGTTTCAGAAACAAGAAAGG
  rat TTCTTTCCTTTGTATCTCTGCACCGCACTCATGACCTT-ATTTCCTGAATCAACACAATCTGGCAACACGCGATTTCAAAAACAAGAAAGG
human TTCTTTCTTTTGTACCTCCCCAGAGTAACAATGACTCT-ATTTCCTGAA-CCAA---AAATTGCAACACGTGGTCTCAAAAATAAGAAAGG
  dog TTGTTTCTCCTGTACCTCCTCCTGGTGGAAAGGACCCTCATTTGCTGAATCCAA----CACTGCGACACGTGAGCTCAGAAAGAAGAAAGG
```

Figure S4: Identification of potential HSF binding sites in the *mPer2* gene.

The sequence motifs GAANNTTC and TTCNNGAA within *mPer2* gene and flanking sequences are represented by black vertical bars. The exon-intron pattern of *mPer2* pre-mRNA is depicted below the map.

The alignment of two highly conserved regions (a and b on the upper map) is shown. The green boxes correspond to the potential HSF binding sites.
