# Supplementary material for: System-Driven and Oscillator-Dependent Circadian Transcription in Mice with a Conditionally Active Liver Clock
Source: PLoS Biol. 2007 Jan 30;5(2):e34. doi: 10.1371/journal.pbio.0050034 (PMC1783671; doi:10.1371/journal.pbio.0050034)
Supplement: Supplementary file 5 [file pbio.0050034.st001.pdf]

Table S1: PCR primers and probes used in this study

**primer**	**sequence** (5' to 3')
Bcl1-HA-Revα	AGGTGATCACCGCCATGGCTTACCC
Bcl1-downstream-Revα	AGGTGATCATCACTGGGCGTCCACC
TRE-fwd	AGCAGAGCTCGTTTAGTGAACC
TRE-rev	CACTGATAGGGAGTGGTA
pLAP-fwd	TGACAGGCATGGCCTATTGA
pLAP-rev	GGCTGGCCTTGAACTTAGAA
LAPtTAtg-fwd	TCTCACTCGGAAGGACAT
LAP-tTAwt-fwd2	TCTGAGCATGGCCTCTAA
LAPtTA-rev2	GCTGGAGTAAATTTCACAGTG
twdTRE	ACCGTACACGCCTACTCGAC
TREvalphaWT2	AGCACCACTGTGAGTTGGTT
TREvalpha-rev2	CCAGTCTCTGGAACATTCTC
TaqMan Bmal1-fwd	CCAAGAAAGTATGGACACAGACAAA
TaqMan Bmal1-rev	GCATTCTTGATCCTTCCTTGGT
TaqMan Bmal1-probe	FAM-TGACCCTCATGGAAGGTTAGAATATGCAGAA-TAMRA
TaqMan Cry1-fwd	CTGGCGTGGAAGTCATCGT
TaqMan Cry1-rev	CTGTCCGCCATTGAGTTCTATG
TaqMan Cry1-probe	FAM-CGCATTTCACATACACTGTATGACCTGGACA-TAMRA
TaqMan Cry2-fwd	TGTCCCTTCCTGTGTGGAAGA
TaqMan Cry2-rev	GCTCCCAGCTTGGCTTGA
TaqMan Cry2-probe	FAM-CAGTCACCCTGTGGCAGAGCCTGG-TAMRA
TaqMan Dbp-fwd	CGTGGAGGTGCTTAATGACCTTT
TaqMan Dbp-rev	CATGGCCTGGAATGCTTGA
TaqMan Dbp-probe	FAM-AACCTGATCCCGCTGATCTCGCC-TAMRA
TaqMan Per1-fwd	ACCAGCGTGTCATGATGACATAC
TaqMan Per1-rev	CTCTCCCGGTCTTGCTTCAG
TaqMan Per1-probe	FAM-CCGTCCAGGGATGCAGCCTCTG-TAMRA
TaqMan Per2-fwd	ATGCTCGCCATCCACAAGA
TaqMan Per2-rev	GCGGAATCGAATGGGAGAAT
TaqMan Per2-probe	FAM-ATCCTACAGGCCGGTGGACAGCC-TAMRA
TaqMan mREV-ERBα-fwd	CATGGTGCTACTGTGTAAGGTGTGT
TaqMan rREV-ERBα-fwd	CATGGTGCTACTGTGTAAAGTGTGT
TaqMan mREV-ERBα-rev	CACAGGCGTGCACTCCATAG
TaqMan rREV-ERBα-rev	CGCAGGCATGCACGCCATAG
TaqMan REV-ERBα-probe	FAM-ACGTGGCCTCAGGC-MGB
TaqMan tbp-fwd	TTGACCTAAAGACCATTGCACTTC
TaqMan tbp-rev	TTCTCATGATGACTGCAGCAAA
TaqMan tbp-probe	FAM-TGCAAGAAATGCTGAATATAATCCCAAGCG-TAMRA
